# Peripherally-derived LGI1-reactive monoclonal antibodies cause epileptic seizures *in vivo*

**DOI:** 10.1093/brain/awae129

**Published:** 2024-04-25

**Authors:** Manoj Upadhya, Toni Kirmann, Max A Wilson, Christian M Simon, Divya Dhangar, Christian Geis, Robyn Williams, Gavin Woodhall, Stefan Hallermann, Sarosh R Irani, Sukhvir K Wright

**Affiliations:** Institute of Health and Neurodevelopment, School of Health and Life Sciences, Aston University, Birmingham, B4 7ET, UK; Faculty of Medicine, Carl-Ludwig-Institute of Physiology, Leipzig University, Leipzig 04103, Germany; Institute of Health and Neurodevelopment, School of Health and Life Sciences, Aston University, Birmingham, B4 7ET, UK; Faculty of Medicine, Carl-Ludwig-Institute of Physiology, Leipzig University, Leipzig 04103, Germany; Institute of Health and Neurodevelopment, School of Health and Life Sciences, Aston University, Birmingham, B4 7ET, UK; Department of Neurology, Section Translational Neuroimmunology, Jena University Hospital, Jena 07747, Germany; Oxford Autoimmune Neurology Group, Nuffield Department of Clinical Neurosciences, University of Oxford, Oxford, OX3 9DU, UK; Departments of Neurology and Neuroscience, Mayo Clinic, Jacksonville, FL 32224, USA; Institute of Health and Neurodevelopment, School of Health and Life Sciences, Aston University, Birmingham, B4 7ET, UK; Faculty of Medicine, Carl-Ludwig-Institute of Physiology, Leipzig University, Leipzig 04103, Germany; Oxford Autoimmune Neurology Group, Nuffield Department of Clinical Neurosciences, University of Oxford, Oxford, OX3 9DU, UK; Departments of Neurology and Neuroscience, Mayo Clinic, Jacksonville, FL 32224, USA; Institute of Health and Neurodevelopment, School of Health and Life Sciences, Aston University, Birmingham, B4 7ET, UK; Department of Neurology, Birmingham Women’s and Children’s Hospital NHS Trust, Birmingham, B4 6NH, UK

**Keywords:** LGI1-Ab encephalitis, faciobrachial dystonic seizures (FBDS), autoimmune-associated epilepsy, seizures, k_v_1 1

## Abstract

One striking clinical hallmark in patients with autoantibodies to leucine-rich glioma inactivated 1 (LGI1) is the very frequent focal seizure semiologies, including faciobrachial dystonic seizures (FBDS), in addition to the amnesia. Polyclonal serum IgGs have successfully modelled the cognitive changes *in vivo* but not seizures. Hence, it remains unclear whether LGI1-autoantibodies are sufficient to cause seizures.

We tested this with the molecularly precise monoclonal antibodies directed against LGI1 [LGI1-monoclonal antibodies (mAbs)], derived from patient circulating B cells. These were directed towards both major domains of LGI1, leucine-rich repeat and epitempin repeat, and infused intracerebroventricularly over 7 days into juvenile male Wistar rats using osmotic pumps. Continuous wireless EEG was recorded from a depth electrode placed in hippocampal CA3 plus behavioural tests for memory and hyperexcitability were performed. Following infusion completion (Day 9), post-mortem brain slices were studied for antibody binding and effects on Kv1.1.

The LGI1-mAbs bound most strongly in the hippocampal CA3 region and induced a significant reduction in Kv1.1 cluster number in this subfield. By comparison to control-Ab injected rats video-EEG analysis over 9 days revealed convulsive and non-convulsive seizure activity in rats infused with LGI1-mAbs, with a significant number of ictal events. Memory was not impaired in the novel object recognition test.

Peripherally-derived human LGI1-mAbs infused into rodent CSF provide strong evidence of direct *in vivo* epileptogenesis with molecular correlations. These findings fulfill criteria for LGI1-antibodies in seizure causation.

## Teaser

Upadhya *et al*. show that antibodies against LGI1 from patients with limbic encephalitis cause seizures in a laboratory model. This is the first time that seizures—as opposed to solely cognitive changes—have been recapitulated in a model of the disorder, adding to evidence for a causal role of LGI1 antibodies in seizure generation.

## Introduction

Autoantibodies to leucine-rich glioma inactivated 1 (LGI1) are identified mainly in older males with a variety of distinctive and frequent focal seizure semiologies, including faciobrachial dystonic seizures (FBDS), piloerection and thermal seizures. In addition, many of these patients develop profound amnesia.^1,2^ FBDS are characterized by brief and numerous seizures characterized predominantly by contractions of the arm and ipsilateral face.^3^ If unrecognised and untreated, FBDS can evolve into a limbic encephalitis (LE) with temporal lobe and tonic-clonic seizures,^3,4^ whereas, early treatment of FBDS with immunotherapies typically dramatically reduces seizure frequencies and can prevent the development of LE, thereby avoiding additional disability.^4^

Medial temporal lobe changes on MRI are a common finding in patients with LGI1-antibodies, and the subsequent atrophy predominantly affects the CA3 region.^5^ Ictal EEG changes have been inconsistently reported, however, the most commonly recognised patterns are generalised attenuation, electrodecremental changes,^2,6,7^ simultaneous frontal EEG and EMG unilateral slow and infraslow wave preceding the contralateral tonic-dystonic seizure,^8^ as well as frequent ‘subclinical’ temporal lobe seizures.^2,8,9^

Polyclonal patient serum-derived IgGs and LGI1-reactive monoclonal antibodies cause neuronal hyperexcitability and even epileptiform activity *in vitro,* with AMPAR and/or Kv1.1 downregulation postulated as the most likely underlying molecular mechanisms.^10,11^ However, despite seizures being a key clinical hallmark of this disease, animal models using LGI1-antibodies have failed to recapitulate this pathognomonic feature *in vivo*.^12,13^ This observation calls into question the direct epileptogenicity of LGI1-antibodies, particularly as the use of polyclonal human serum means that non-LGI1 reactivities may mediate observed outcomes.

Here, we use human-derived monoclonal antibodies with specific reactivities to either the leucine-rich repeat (LRR) or the epitempin repeat (EPTP) domain of LGI1,^14^ to produce a passive transfer rodent model with spontaneous epileptic seizures and video-EEG recorded ictal changes.

## Materials and methods

### Antibody preparation

Two LGI1-specific monoclonal antibodies (mAbs), targeting either the LRR (mAb2) or EPTP (mAb13) domains were produced, as described previously.^14^

### *In vivo* experiments

#### Animals

**Figure 1:**
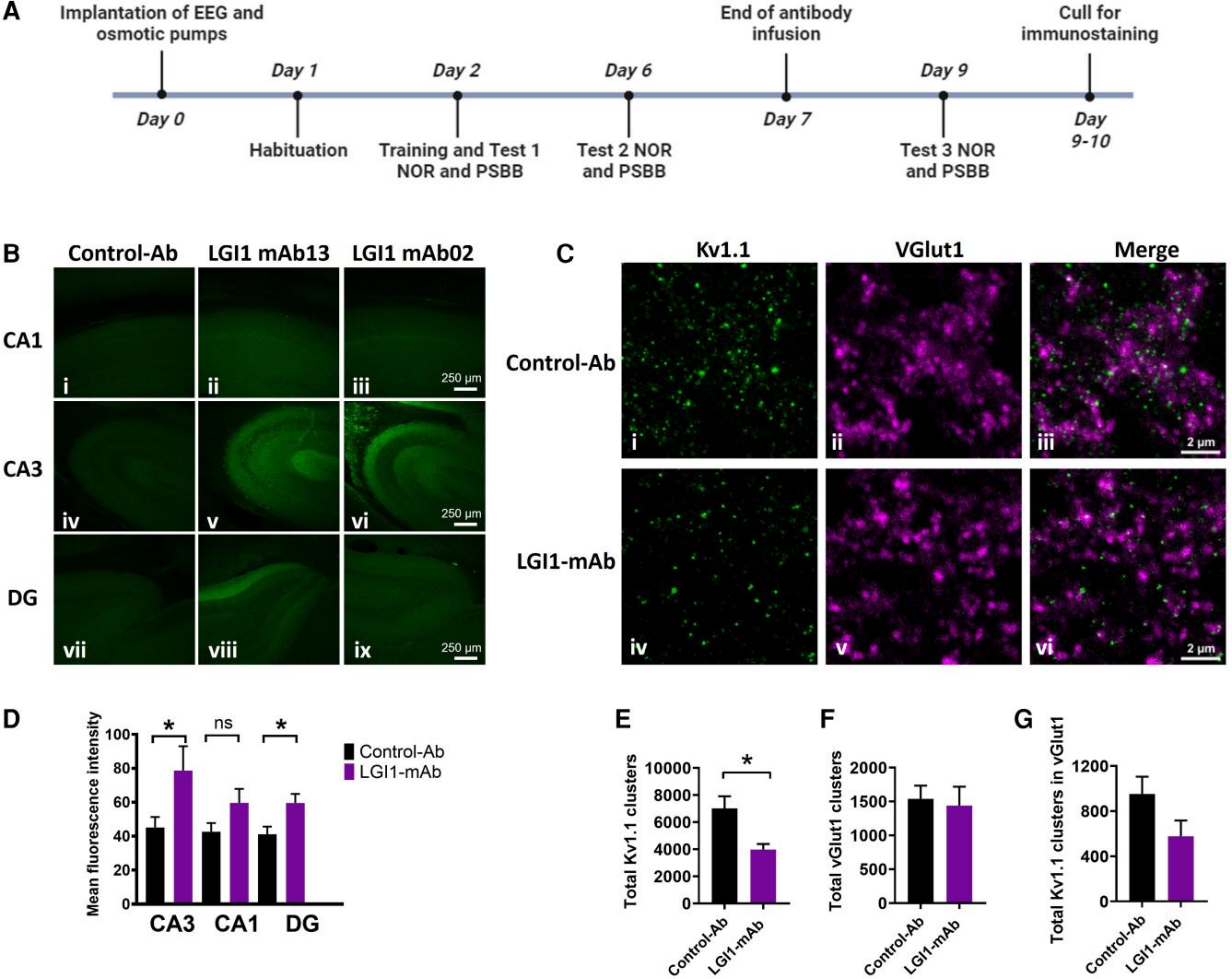
**Patient derived monoclonal LGI1 antibodies bind most strongly to CA3 region of the hippocampus and cause a decrease of total synaptic clusters of kv1.1 following 7-day intracerebroventricular infusion**. (**A**) Experimental timeline. [**B**(**i**–**ix**)] Representative confocal images of hippocampus [CA1 (**i**–**iii**), CA3 (**iv**–**vi**) and dentate gyrus (DG; **vii**–**ix**)] from sagittal brain slice prepared after 7 days of infusion of control-antibody (control-Ab; *n* = 6; **i**, **iv** and **vii**) and LGI1-monoclonal antibodies (mAbs) directed against epitempin repeat (EPTP; *n* = 3; **ii**, **v** and **viii**) and leucine rich-repeat (LRR; *n* = 4; **iii**, **vi** and **ix**) epitopes shows typical staining pattern with secondary anti-human IgG (green). Scale bar = 250 µm. [**C**(**i**–**vi**)] Example images of stimulated emission depletion microscopy of a section from the CA3 region stained for Kv1.1 (*left*) and VGlut1 (*middle*) and merged (*right*) from brains treated with control-Ab (*top*) or LGI1-mAbs (*bottom*). (**D**) LGI1 mAb-treated brain slices (17 slices from seven animals; *n* = 5 images of CA3, CA1 and DG regions from each slice; mean fluorescence intensity log EC50 values compared with control-Ab (21 brain slices from six animals; *n* = 5 images of CA3, CA1 and DG regions from each slice). *P < 0.05, Mann–Whitney. (**E**) Quantification of density of total Kv1.1 clusters in pooled analysis of CA3 region in animals treated with control or LGI mAbs. (**F**) Quantification of density of total vGlut1 clusters in pooled analysis of CA3 region in animals treated with control or LGI mAbs. (**G**) Quantification of total KV1.1 clusters colocalizing with vGlut1. (**D**–**G**) From *n* = 5 images of CA3 region for each brain sample from six control-Ab infused and seven LGI1-mAb infused rodents (four LRR-specific and three EPTP-specific). Data are presented as mean ± standard error of the mean. *P < 0.05, **P < 0.01, ***P < 0.001.

Sixteen postnatal Day 21 (P21) male Wistar rats, weighing 52–60 g were used for the experiments described. Animals were housed in temperature- and humidity-controlled conditions with a 12 h/12 h light/dark cycle and allowed free access to food and water. All procedures were compliant with current UK Home Office guidelines as required by the Home Office Animals (Scientific Procedures) Act 1986 and carried out under the authority and procedural approval of a UK Home Office approved project license and in line with ARRIVE guidelines. The Aston Bioethics Committee, University of Aston, Birmingham, UK granted local ethical approval for the study. Full experimental timeline shown in Fig. 1A.

### EEG implantation and osmotic pump surgery

The rats were implanted, as previously described, with subcutaneous transmitters for *in vivo* EEG recordings [A3028B-DD subcutaneous transmitters, 90-mm leads, OpenSource Instruments (OSI)] via unilateral depth electrode [W-Electrode (SCE-W), OSI] in left hippocampus (CA3, 3.5 mm lateral, 3.8 mm caudal, depth 2.3 mm), and subcutaneous osmotic pumps (model 1007D, Azlet) (volume 100 µl, flow rate 0.5 µl/h, duration 7 days, primed overnight) attached to bilateral cannulae (328OPD-3.0/SPC, PlasticsOne), implanted into the lateral ventricles (±1.5 mm lateral and 0.6 mm caudal).^15-17^ A reference EEG electrode was implanted on the contralateral skull surface (3.5 mm lateral, 3.8 mm caudal); the cannula and skull electrodes were secured with dental cement.^15,16^

### EEG data analysis

EEG data (wide band pass 0.2–160 Hz sampled at 512 samples per second) was collected and recorded using Neuroarchiver software (OSI) from wireless transmitters implanted in freely moving animals placed in a custom-built Faraday cage with aerial (OSI) as previously described.^15-17^ For automated ictal event detection, video-EEG matching was used to identify ictal EEG events. The Event Classifier (OSI) was used to classify segments (1 s) of EEG according to program metrics (power, coastline, intermittency, coherence, asymmetry, spikiness) creating clusters of similar events when plotted. This generated a library of ictal events that allowed fast identification of abnormal EEG events by automated comparison (http://www.opensourceinstruments.com/Electronics/A3018/Seizure_Detection.html). Powerband analysis was carried out using a custom-designed macro.

### Behavioural testing

#### Novel object recognition

The novel object recognition (NOR) test is used to test cognitive performance in autoimmune encephalitis models.^13,14^ Animals were habituated to the test arena 24 h before training and tests. The time interacting with the objects was measured and the NOR index (time interacted with novel object/total time of interaction with both the objects) calculated. Three tests were carried out at regular intervals during the 9-day recording period. All the observations were carried out by the experimenter blind to the treatments and analysed using the Ethovision software. The frequency of rats to approach the object, time spent with the objects was measured for NOR index, and the distance travelled, and velocity was measured as a measure of anxiety score.

#### Post-Seizure Behavioural Battery

The Post-Seizure Behavioural Battery (PSBB) was performed as previously described to assess hyperexcitability and other behavioural indices suggestive of epilepsy.^16,18^ Two simple and non-stressful tasks, ‘touch’ and ‘pick-up’ tasks, constituted the PSBB and were performed at three time points. PSBB scores were calculated by taking the product of the task scores (‘Touch × Pick-up’).

### Local field potential recordings

Local field potential (LFP) recordings were performed and analysed as previously described.^15,16^ Briefly, on Day 9 immediately after the behavioural experiments, rats were anaesthetized using isoflurane and following the loss of consciousness, pentobarbital (60 mg/kg, subcutaneous) and xylazine (10 mg/kg, intramuscular) injected. Transcardial perfusion was then performed using ice-cold artificial CSF (aCSF). Animals were decapitated, and the brain was removed. Brain slices were prepared using a vibratome (Campden Instruments) at 450 μm for LFP recording and 350 μm for fluorescence intensity measurements. LFP recordings were assessed for spike activity using Spike2 software (CED). Root mean square (RMS) amplitude of each recording was calculated, and events with an amplitude greater than 5-fold the RMS amplitude were considered a ‘spike’.

### Immunofluorescence, immunohistochemistry and image analysis

The immunofluorescence study was performed as previously reported.^15,16^

Further detailed methods are available in the Supplementary material.

### Statistical analysis

For behavioural experiments, repeated-measures two-way ANOVA with Bonferroni multiple comparison tests and non-parametric Mann–Whitney T-tests were used to assess differences. For *in vivo* experiments and immunohistochemistry, two-tailed Mann–Whitney T-tests were used. *P* < 0.05 was considered as significant. Statistical analysis was conducted using GraphPad Prism 8 (GraphPad Software Inc).

## Results

### LGI1 antibodies bind to rat hippocampus and cause reduction of kv1.1 clusters

Seven-day osmotic pumps delivered the LGI1-mAbs (*n* = 10; five animals with LRR and five with EPTP specific mAbs) and control Abs (*n* = 6) into the lateral cerebral ventricles of P21Wistar rats.

After interventricular infusion, both LGI1-mAbs (LRR- and EPTP-specific) bound to rodent hippocampus (Fig. 1B), confirming previous studies in mice.^14^ By comparison to the control, non-brain-reactive Abs, the fluorescence intensities of bound LGI1-mAbs were highest in the CA3 and dentate gyrus (Fig. 1C and Supplementary Fig. 1A) This increased binding was also seen in the contralateral hemisphere (without depth electrode; Supplementary Fig. 1B). As demonstrated with prior use of these antibodies, the LRR mAbs were strongly retained in the infused hippocampus; EPTP showed slightly less residual binding overall on both sides (Supplementary Fig. 1A and B).^14^ In the CA3 region, super-resolution light microscopy using stimulated emission depletion (STED) of 20-µm-thick cryosections revealed that the number of Kv1.1 clusters was significantly lower in the LGI1-mAb infused group (pooled analysis of LRR and EPTP) compared with the controls (Fig. 1D and E), and non-significantly trended to those which co-localized with the glutamatergic synapse marker vGlut1 (Fig. 1F and G).

### LGI1-mAbs cause clinical and subclinical spontaneous epileptic seizures *in vivo*

**Figure 2:**
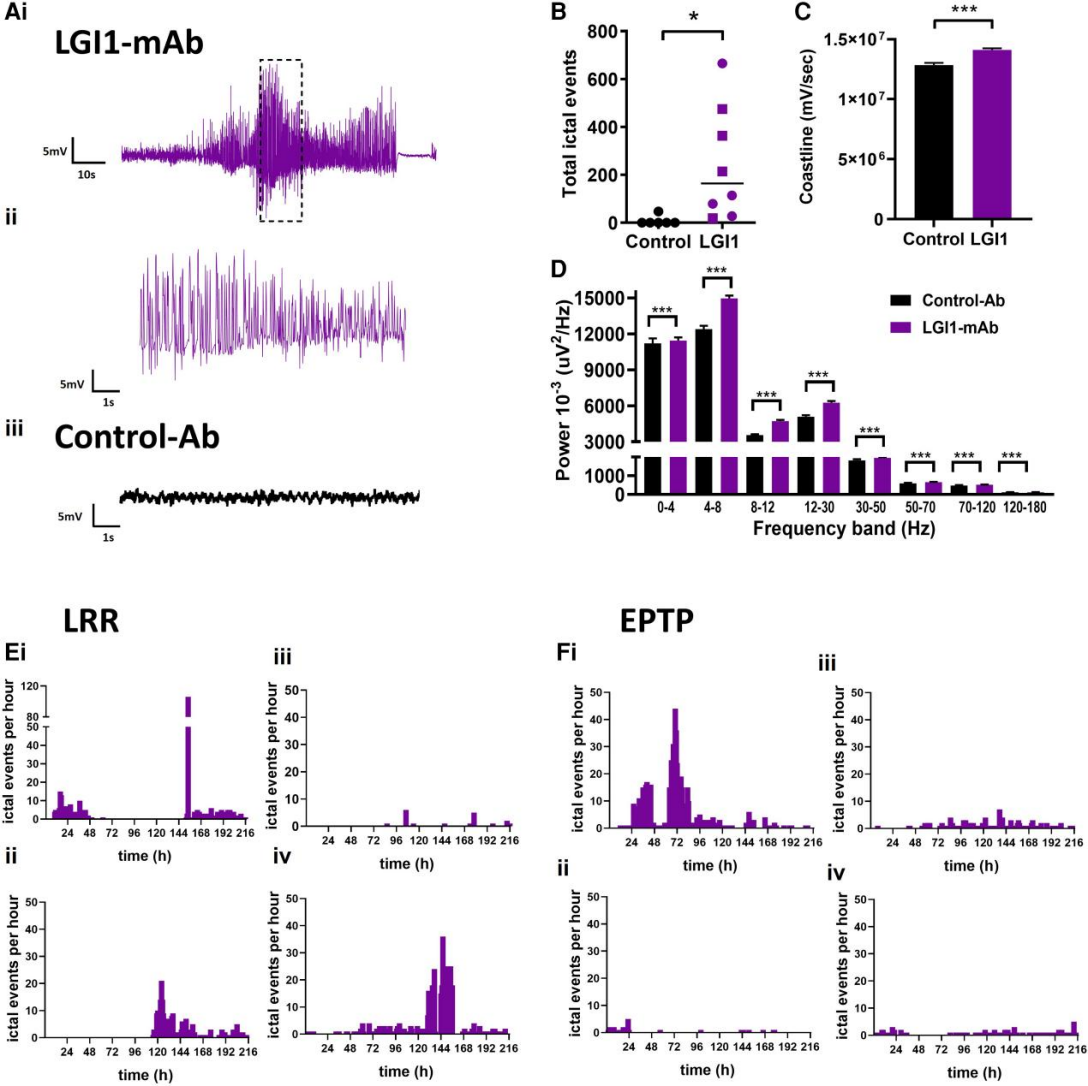
**Intracerebroventricular infusion (7 days) of patient derived monoclonal LGI1 antibodies induces seizures and epileptiform activity *in vivo*.** [**A**(**i**–**iii**)] Example EEG recording from rodent using wireless EEG transmitter during intracerebroventricular infusion of LGI-monoclonal antibody (mAb) directed towards leucine rich-repeat (LRR) epitope (Supplementary Video 1). (**i**) Highlighted/hatched EEG area expanded in **ii**; control-Ab infused rodent EEG shown for comparison in **iii**. (**B**) Total number of 1-s ictal events recorded during 7-day intracerebroventricular infusion of LGI-mAbs (*n* = 8; four LRR and four epitempin repeat (EPTP) epitopes] compared with control-Ab infused animals (*n* = 6). Squares represent rodents infused with LRR epitope-specific LGI1-mAb, circles represent EPTP epitope-specific LGI1-mAb. Data are presented as mean ± standard error of the mean. (Mann–Whitney, *P < 0.05). (**C**) Averaged hourly EEG coastline length for LGI1-mAb infused rodents (*n* = 8; four LRR epitope and four EPTP) compared with controls (*n* = 6) (Mann–Whitney, ***P < 0.001). (**D**) Hourly EEG power averages over 7-day infusion and recording period of LGI1-mAbs versus controls (Mann–Whitney, ***P < 0.001; rodent numbers as in **B** and **C**). [**E**(**i**–**iv**)] Plots of frequency, duration and recurrence of ictal events in each animal infused with LGI1-mAbs directed towards the LRR epitope. Video-EEG analysis revealed tonic-clonic seizures (Supplementary Video 2), non-convulsive ictal events (**i**, **ii** and **iii**), myoclonic jerks and wet-dog shakes (**iv**) (Supplementary Video 3). [**F**(**i**–**iv**)] Plots of frequency, duration and recurrence of ictal events in each animal infused with LGI1-mAbs directed towards the EPTP epitope. Video-EEG analysis revealed tonic-clonic seizures(**i**), non-convulsive ictal events (**i**–**iv**) and myoclonic jerks (**iii**).

To investigate potential epileptogenic effects, brain activity was recorded continuously during LGI1-mAb infusion using wireless EEG transmitters from a depth electrode placed in the CA3 region of hippocampus. One rodent infused with EPTP specific LGI1-mAb was culled early due to excess lacrimation, and in one rodent infused with LRR specific mAb, the EEG recording was suboptimal for analysis but the animal data were still included for behavioural testing and post-mortem immunohistochemistry. There was no mortality or other significant morbidity following seizures and documented welfare scores remained low throughout the experimental period indicating minimal pain or distress. There was ictal activity recorded in one control animal within 12 h of surgery, most likely due to acute effects of surgery/anaesthesia. The PSBB score remained <2 for this animal throughout the experimental period with no further ictal activity noted on EEG. In the LGI1-mAb infused rats, the frequency, duration and recurrence of ictal events as identified through automated seizure detection^15-17^ in each animal treated is shown in Fig. 2E and F. The recorded epileptic and behavioural activity included convulsive and non-convulsive ictal events (Fig. 2A, E and F and Supplementary Videos 1–3). We observed a significantly higher total number of ictal events in the LGI1-mAb infused animals compared to controls during the 9 day EEG recording period (Fig. 2B). The EEG coastline was higher in LGI1-mAb infused animals, due to higher prevalence of relatively high-amplitude epileptiform events (Fig. 2C). This cohort also showed higher power in all EEG frequency bands. Similar spectral power changes compared to controls are seen in other autoimmune-encephalitis-associated seizure models and models of status epilepticus, reflecting the presence of recurrent spontaneous convulsive and non-convulsive seizures^19-22^ (Fig. 2D). Following completion of the antibody infusion, LGI1-mAb and control-Ab infused animals were terminally anaesthetized and brains extracted for acute *in vitro* brain slice recording. Local field potentials were recorded in CA3 and CA1 of the hippocampus, spike activity was not significantly different from control slices (Supplementary Fig. 1C). This is concordant with the minimal *in vivo* ictal activity recorded by Day 9 (Fig. 2E and F).

### LGI1-mAbs cause enhanced startle response and aggressive behaviour without affecting cognition

**Figure 3:**
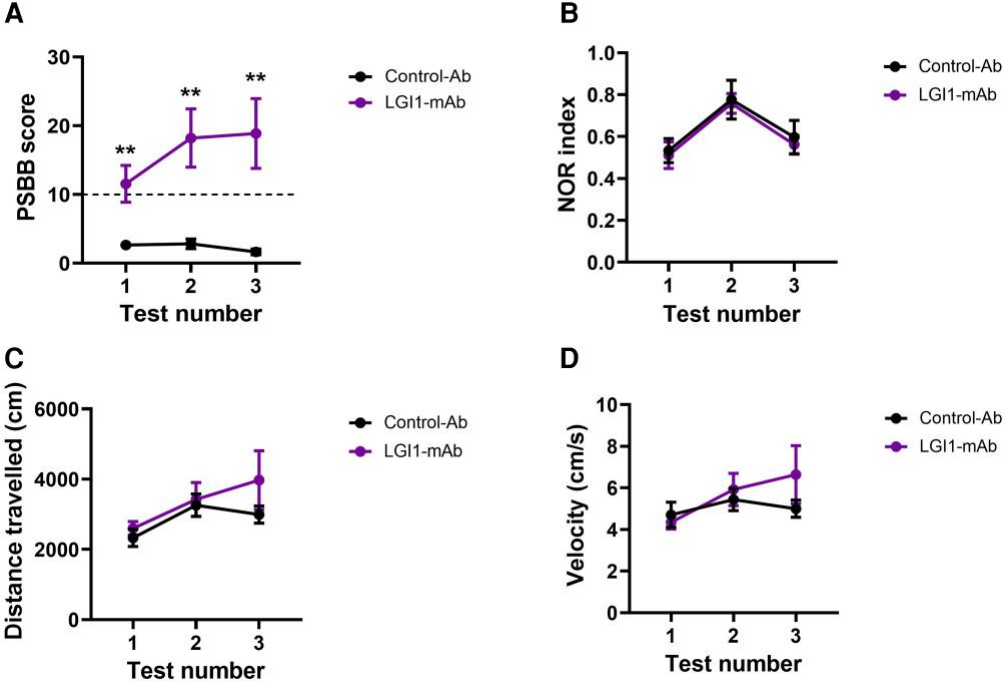
**Intracerebroventricular infusion (7 days) of patient-derived monoclonal LGI1 antibodies causes enhanced startle response and aggression but has no effect on memory in behavioural tests**. (**A**) Post-seizure behavioural battery (PSBB) scores in rodents over 7-day intracerebroventricular infusion of LGI-monoclonal antibodies (mAbs) [*n* = 9; five leucine-rich repeat (LRR) and four epitempin repeat (EPTP)] and control-Ab (*n* = 6) (one-way ANOVA, **P < 0.01); dotted line represents a score of 10, over this is considered suggestive of behaviour consistent with spontaneous recurrent seizures. (**B**) Novel object recognition (NOR) index calculated at three time points of LGI1-mAbs (*n* = 7; four LRR and three EPTP) and control-Ab (*n* = 6) infused rats. (**C**) Distance travelled by rats and (**D**) velocity measured during NOR tests in rats infused with control-Ab (*n* = 6) and LGI1-mAbs (*n* = 7; four LRR and three EPTP) infused rats. Data are presented as mean ± standard error of the mean.

The PSBB was used to monitor behavioural changes (enhanced startle response and aggression) consistent with the development of spontaneous recurrent seizures.^16,18^ Animals were tested three times over 9 days using touch and pick-up tests, with a score > 10 indicating significant behavioural change. The LGI1-mAb infused animals scored significantly highly at all three time-points compared to the control-Ab infused animals where the scores remained consistently low (Fig. 3A and Supplementary Fig. 1).There were no differences in the NOR index (calculated by dividing the time spent with the novel object with the entire duration of time spent with both the objects), locomotion, and distance travelled and velocity, during NOR across three time points (Day 1, Day 5 and Day 9; Fig. 3B–D and Supplementary Fig. 1).

## Discussion

LGI1-antibody encephalitis patients exhibit focal and tonic-clonic seizures as well as cognitive impairment. Published animal models were based on application of serum-IgGs to successfully model the cognitive changes *in vivo,* but seizures were not seen.^12,13^ Here, we used highly specific patient-derived LGI1-mAbs, which lack the potential additional reactivities potentially present in serum. After infusion into the CSF of juvenile Wistar rats, these LGI1-mAbs led to seizures *in vivo*. Similar to patient phenotypes, where epileptiform discharges are the most frequent EEG finding,^19^ we recorded clinical and subclinical seizures, with ictal EEG changes. These findings support the direct epileptogenicity of LGI1 antibodies and allow them to fulfill Wietebsky’s postulates of pathogenicity.^20^ With continuing controversy over whether LGI-Ab associated seizures are in fact a paroxysmal movement disorder and not ictal,^21^ this study confirms the causal link between the molecular interactions affecting Kv1.1 mediated by LGI1-Abs and seizure activity *in vivo*.

LGI1 is a secreted neuronal protein that interacts with the ADAM22 and ADAM23 transmembrane metalloproteases forming a trans-synaptic protein complex that facilitates excitatory synaptic transmission.^22^ LGI1 consists of an N-terminal LRR domain and a C-terminal EPTP domain. The LRR-directed monoclonal antibody used in this study binds the ADAM22/23-docked LGI1 complex *in vitro*, causing internalisation and hence disruption of the membrane stabilising trans-synaptic complex.^14^ This pathomechanism is similar to that demonstrated by a CSF-derived LGI1-specific mAb that also caused epileptiform changes *in vitro.*^10^*In vitro* primary neuronal cultures also incubated for 7 days with LRR- and EPTP-specific mAbs showed that LRR-directed mAbs could directly affect neuronal excitability, but this effect was less pronounced in EPTP-specific mAb exposed neurons.^23^ In contrast to the action of LRR mAbs *in vitro*, the EPTP mAbs are reported to exert their effects by *directly* inhibiting the docking of LGI1 to the ADAM proteins.^14^ In our study, EEG recordings have demonstrated increases in network and neuronal excitability in hippocampal CA3 *in vivo* by both LRR and EPTP domain directed LGI1 autoantibodies.^10,23^ Studies have shown that LRR- and EPTP-specific antibodies co-occur in patients with LGI1-antibody encephalitis but the numbers of recorded rats with either mAb subclass do not allow for statistical comparison of epitope-specific clinical features (Supplementary Fig. 1).^14^ The goal of future studies is to analyse our *in vivo* EEG using computational modelling to evaluate different hypotheses pertaining to network hyperexcitability and seizures by action of epitope specific LGI1-antibodies and to differentiate epitope-specific effects.^15,24^

EPTP specific mAbs are reported to have no effect on cognitive performance when injected into hippocampi of mice^14^: this may explain why our pooled NOR results from both LRR and EPTP specific mAbs infused rats were unremarkable. Additionally, in contrast to previous *in vivo* passive transfer models, our antibody infusion time was much shorter: 7 days, compared with 14 days. Patients with LGI1-Ab encephalitis frequently develop seizures before the onset of memory disturbance^25^ and this could explain our behavioural findings in this rodent model. Lastly, the genetic background of rodents can impact on seizure susceptibility^12,17,26^; here juvenile Wistar rats were used as they have proven to be effective in modelling autoimmune-associated seizures and epilepsy *in vivo*.^15,16^

The goal of this study was to prove epileptogenicity of LGI1 mAbs *in vivo* using mAbs with known pathogenic effects *in vitro*. We also took this opportunity to generate some insights into identifying a pre- or post- synaptic mechanism for seizure generation. Previous longer-term infusion (14 days) *in vivo* studies using patient-derived LGI1-mAbs that target both the LRR- and EPTP domains did show a disruption in presynaptic and postsynaptic LGI1 signalling.^13^ This was due to an initial decrease of Kv1 levels (at 13 days) followed by AMPA receptor downregulation by 18 days post-infusion.^13^ We also demonstrated loss of Kv1.1 clusters in hippocampal CA3 (with a trend towards a reduction in glutamatergic synapses) but our infusion time was shorter (7 days), and longer-term time points were not analysed. We chose to record our EEG from the hippocampus as the expression pattern of the LGI1 protein in rats shows a prominent distribution here^27^ and LGI1 antibodies are known to bind avidly to the CA3 area hippocampal subfield in murine sections.^1^ Additionally, subacute reduction of hippocampal LGI1 with alternative methods (shRNA) also increases neuronal network excitability.^28^ In patients with LE and LGI-Abs, bilateral focal CA3 hippocampal subfield atrophy has also been described, and the temporal lobe is the area where epileptiform activity is most frequently located.^5,19^ The primary motor cortex has also recently been identified as an additional target for LGI antibodies.^8^ Our study did not record from motor cortex however a recent elegant study using injections of dendrotoxin to block Kv1.1 channels in the rodent motor cortex faithfully recapitulated EEG changes and the clinical phenotype of tonic-dystonic seizures seen in patients with LGI1-Abs.^29^ This alternative approach to passive transfer i.e. targeting the pathophysiological mechanisms downstream to LGI1, through a direct pharmacological inhibition of Kv1.1 channels, is a promising and exciting area in antibody-mediated seizure model development. Interestingly, although tonic-dystonic seizures were not observed in the current study, some of the clinical characteristics (e.g. behavioural change, orofacial seizures) did overlap with spontaneously arising feline LGI1-autoantibody limbic encephalitis described in a recent large international cohort.^30^

In summary, in this focused study, we have developed a passive transfer *in vivo* model of LGI1-mAb associated seizures which displays epileptiform activity in keeping with the human disease. The use of wireless EEG telemetry and continuous video-EEG allows for accurate tracking of behavioural and EEG changes. Future studies will focus on interrogating new and existing data on exact pathomechanisms for *in vivo* epileptogenicity and identification of possible novel epitope-specific treatment targets.

## Supplementary Material

awae129_Supplementary_Data

## Data Availability

Data are available on request.
